# Abnormal Cortical Activation Patterns Among Chinese-Speaking Schizophrenia Patients During Category and Letter Verbal Fluency Tasks Revealed by Multi-Channel Functional Near-Infrared Spectroscopy

**DOI:** 10.3389/fpsyt.2021.790732

**Published:** 2021-11-25

**Authors:** Juan Li, Junlin Mu, Chenyu Shen, Guanqun Yao, Kun Feng, Xiaoqian Zhang, Pozi Liu

**Affiliations:** ^1^School of Clinical Medicine, Tsinghua University, Beijing, China; ^2^Department of Psychiatry, Tsinghua University Yuquan Hospital (Tsinghua University Hospital of Integrated Traditional Chinese and Western Medicine), Beijing, China; ^3^Department of Neuroelectrophysiology, The Second Affiliated Hospital of Xinxiang Medical University, Xinxiang, China

**Keywords:** functional near-infrared spectroscopy, fNIRS, category fluency task, letter fluency task, schizophrenia

## Abstract

**Background:** Functional near-infrared spectroscopy (fNIRS) has many advantages over other neuroimaging modalities for routine measurement of task-dependent cortical activation, but most fNIRS studies of schizophrenia have used letter fluency tasks (LFTs). Further, performances on category fluency tasks (CFTs) and LFTs may be distinct in Chinese patients due to the unique semantic features of Chinese written characters. To identify unique disease biomarkers measurable by fNIRS in Chinese schizophrenia patients, this study compared cortical oxygenated hemoglobin changes ([oxy-Hb]) during a Chinese LFT and CFT between patients and healthy controls.

**Methods:** Inpatients of the Second Affiliated Hospital of Xinxiang Medical University were recruited from Match 2020 to July 2021. The Positive and Negative Symptom Scale (PANSS) was used to evaluate psychiatric symptoms. Dynamic changes in [oxy-Hb], an indicator of neural activity, were measured during CFT and LFT performance by 52-channel fNIRS.

**Results:** Forty-seven schizophrenia inpatients and 29 healthy controls completed all tests. Schizophrenia patients showed significant cortical activation at 15 channels covering the left hemisphere and 17 channels over the right hemisphere during the CFT. During the LFT, activity was significantly increased at only six channels, all over the left hemisphere (FDR *P* < 0.05). In healthy controls, significant [oxy-Hb] increases were found at 24 channels over the left hemisphere and 19 channels over the right hemisphere during CFT. While during the LFT, the significant increases were found at 7 channels all over the left hemisphere (FDR *P* < 0.05). When years of education was included as a covariate, the schizophrenia group demonstrated no significant hypoactivation relative to healthy controls at any channel after FDR correction (FDR *P* < 0.05) during CFT while demonstrated significant hypoactivation at channel 11 during LFT (FDR *P* < 0.05). There were no significant associations between PANSS scores and [oxy-Hb] changes after FDR correction (FDR *P* < 0.05).

**Conclusions:** Left lateralization during CFT was reduced among schizophrenia patients and may be related to the semantic deficit. The Chinese-CFT could be a more sensitive indicator of frontal-temporal dysfunction in schizophrenia.

## Background

Schizophrenia (SP) is a complex disease with heterogeneous symptom expression, unclear etiology, high global disease burden, and generally poor life outcome (1). The global mean prevalence of schizophrenia is nearly 2% (1), but a recent epidemiological study from China (2) reported a 0.6% lifetime prevalence. This gap may be explained by diagnostic inaccuracy due to symptom heterogeneity (3, 4). Hence, diagnosis and patient care in China may benefit from the identification of biomarkers specific for SP and associated deficits.

Cognitive deficits are critical symptoms of SP (5) as they are more persistent than psychotic symptoms, less responsive to currently available drugs, and a better predictor of long-term functional disability (6). Furthermore, cognitive deficits are viewed as an independent symptom of SP and may reflect the underlying psychopathology (5, 7). Verbal fluency tasks (VFTs) are widely used to evaluate cognitive deficits in mental disorders. Two major subtypes of VFTs are letter (phoneme) fluency tasks (LFTs) and category (semantic) fluency tasks (CFTs) (7). In LFTs, subjects must generate words based on phonemic (phonological) characteristics such as the first letter or sound, while in CFTs, subjects must generate words in defined semantic categories (e.g., cities, items used for cooking). Deficient verbal fluency is viewed as a predictor of psychosis (7) but is stable in chronic schizophrenia (8). However, it is still controversial whether lack of semantic or phoneme fluency is the more serious deficit in schizophrenia (9–11). Additionally, most studies on fluency deficits in schizophrenia have employed English or Japanese LFTs (12–15). Unlike other languages, Chinese characters, known as pictographs, contain semantic information, so to elucidate language deficiency in Chinese schizophrenics, it is necessary to conduct studies using carefully constructed Chinese VFTs (12).

Measurement of brain activation patterns during VFT performance may provide clues to the biological mechanism underlying language deficits in schizophrenia and reveal markers for diagnosis, prognosis, and treatment evaluation (16–18). Functional near-infrared spectroscopy (fNIRS) has become a common non-invasive neuroimaging modality for this purpose as it is less invasive, less costly, and yields higher temporal resolution data than magnetic resonance imaging (MRI) (19, 20). Previous studies have shown reduced prefrontal cortex activation in patients with schizophrenia during a VFT, suggesting that fNIRS is a suitable tool for identifying candidate biomarkers (13, 21). Nevertheless, there are still no reliable neuroimaging biomarkers due to the different task designs and patient heterogeneity in previous studies. Furthermore, few combined fNIRS–VFT studies have examined mental disorders in Chinese patients.

Consistent with previous studies in other patient populations (12, 22, 23), reduced activation over the frontotemporal region has been reported in fNIRS studies of Chinese-speaking schizophrenia patients. Alternatively, there have been no comparisons of cortical activity during LFTs and CFTs in Chinese patients. In fNIRS studies of Chinese patients, the LFT of 60 s task period is commonly employed, and Quan et al. (12) reported reduced brain activation in the prefrontal and superior temporal cortices of schizophrenics. Although this 60-s task paradigm allows for easy comparison across studies, this measurement interval may impact NIRS power in block design studies, where the best segment time appears to be 30 s (14). Alternatively, the CFT has been used with 30 s activation time (24, 25). However, no study has directly compared cortical activity during Chinese versions of the CFT and LFT between patients and healthy controls (HCs), so the mechanisms underlying language deficits in Chinese schizophrenics remain unclear.

The current study compared brain activation patterns between schizophrenia patients and HCs in the Chinese population during CFT and LFT performance using 52-channel fNIRS. The primary aim was to identify potential neuroimaging biomarkers and to determine the more sensitive task (CFT or LFT) for evaluation of language deficits in Chinese schizophrenia patients.

## Methods

### Participants

Inpatients of the Second Affiliated Hospital of Xinxiang Medical University (Xinxiang, Henan, China) were recruited from Match 2020 to July 2021. Inclusion criteria were (i) SP diagnosed according to the Diagnostic and Statistical Manual of Mental Disorders, Fifth Edition, (ii) 18–45 years old, (iii) duration of disease <10 years, (iv) no <9 years of education, and (v) right-handed. Exclusion criteria were (i) intellectual disability, a history of neurological diseases, or other serious physical diseases (such as liver/kidney/heart failure), (ii) received electroconvulsive therapy within 2 years, (iii) previous use of typical antipsychotic drugs, (iv) family history of mental disorders, (v) pregnancy, and (vi) a history of drug/substance abuse or addiction (except tobacco). Diagnoses were made by two associate chief physicians in psychiatry. All patients had residual symptoms but were in a relatively stable phase (antipsychotic medication dose stable for more than one week) that enabled completion of the assessment.

Age- and sex-matched HCs were recruited from the community during the same period. All were free of medication and evaluated by an experienced psychiatrist for exclusion of mental illness using the International Neuropsychiatric Interview. Candidate HCs were excluded for a personal or family history of neuropsychiatric illness, drug/substance abuse, addiction (except tobacco), or pregnancy.

### Positive and Negative Symptom Scale (PANSS)

The Chinese version of the PANSS (26) was administered as a semi-structured interview to estimate the severity of schizophrenia symptoms. The Chinese PANSS has demonstrated strong internal consistency (Cronbach's alpha = 0.87). The PANSS administrators were well-trained and demonstrated good inter-rater consistency.

### Verbal Fluency Task

**Figure 1:**
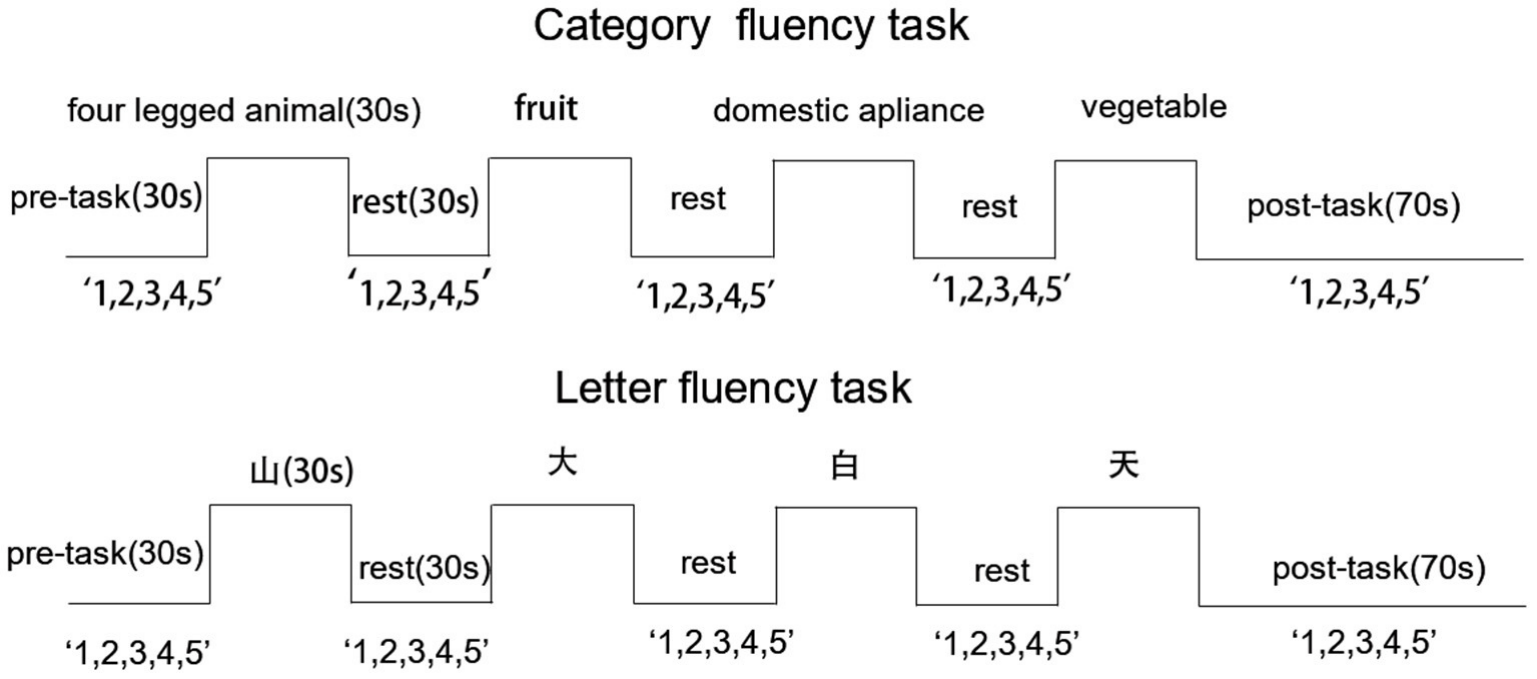
Activation task design. Cues are presented along the top and “1,2,3,4,5” indicates that participants were requested to count from 1 to 5 repeatedly during pre-task, inter-trial, and post-task periods. Excepted for the 70-s post-task period, all other periods were 30 s.

Each participant completed a CFT and LFT in succession. Each test required 310 s and tests were administered in a four block-design (see Figure 1). Both tests were present on a computer screen using E-prime 2.0. Before the formal test, a practice session was conducted to make sure participants understood the task. To ensure a stable fNIRS waveform, a 10-s delay was inserted prior to task onset, followed by a 30-s pre-task period during which participants counted “1, 2, 3, 4, 5” repeatedly until the task began. For each 30-s task period, participants were requested to generate as many words as possible in response to a cue. The CFT cues were “four-legged animal,” “fruit,” “domestic appliance,” and “vegetable,” while cues for the LFT were words beginning with “山,” “大,” “白,” and “天.” There was a 30-s rest period between tasks and the participants were also instructed to count “1, 2, 3, 4, 5” repeatedly during this period. In the 70-s post-task period, the participants were asked to count as in the pre-task period. Participants were instructed to avoid movement, speak quietly, and minimize blinking during the test. Valid words were recorded by a researcher.

### fNIRS Measurements

**Figure 2:**
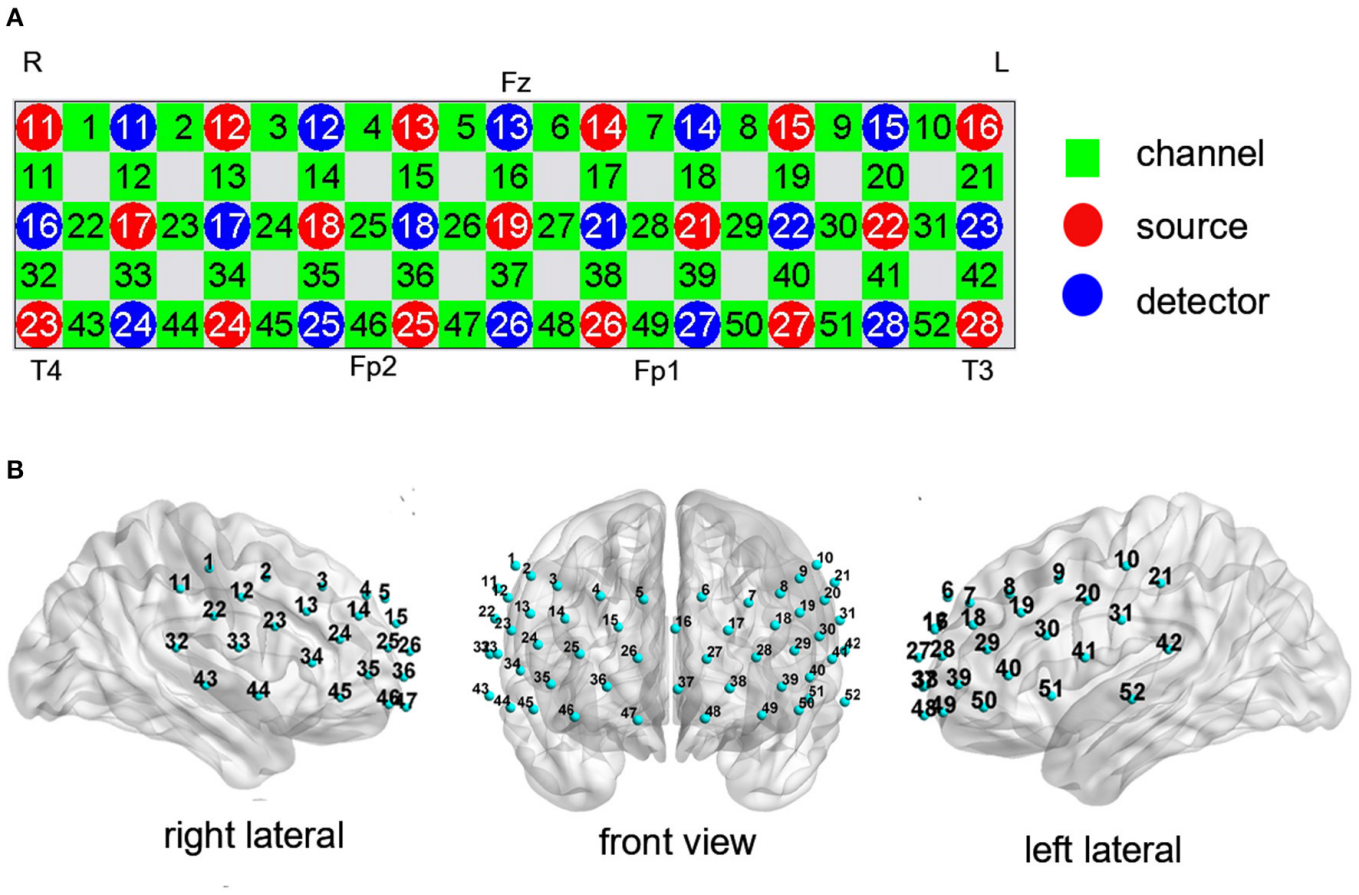
Measurement points of the 52-channel near-infrared spectroscopy (NIRS) system. **(A)** The arrangement of channels was based on the international 10–20 system. **(B)** The 3-dimensional detection region for each channel.

Cortical activation was measured during the CFT and LFT using a 52-channel NIRS system with 695 and 830 nm detectors (ETG-4100, Hitachi Medical Corporation, Tokyo, Japan). Relative changes in oxy-Hb concentration ([oxy-Hb]) were measured according to the modified Beer-Lambert law. The 52-channel system consisted of 17 light emitters and 16 light detectors fixed with 3 ×11 thermoplastic shells (Figure 2A). This array can measure the cortical activation in the frontal and superior temporal cortices based on the international 10–20 system used in electroencephalography (Figure 2B). A channel was defined as the area between light emitters and light detectors (inter-optode distance of 30 mm) and signals were sampled at 10 Hz. The corresponding spatial information for each channel in Montreal Neurological Institute space was evaluated using NIRS_SPM (version 4.0) (27).

### fNIRS Signal Analysis

Previous studies have concluded that [oxy-Hb] is more strongly related to blood oxygenation level-dependent (BOLD) signals as measured by functional MRI (fMRI) than [deoxy-Hb] (28–30). Hence, the current study recorded [oxy-Hb] as a measure of cortical activation during the CFT and LFT. All NIRS data were analyzed using NIRS-SPM (https://www.nitrc.org/projects/nirs_spm/). The hemodynamic response function (HRF) and a Wavelet–minimum description length (MDL)-based detrending method were used to remove systemic noise and physiological variation (31). The false discovery rate (FDR) (32) was applied to correct for multiple comparisons (level set at *P* < 0.05). The visualized brain network was presented using BrainNet Viewer (33).

### Statistical Analysis

SPSS version 25.0 (IBM Corporation, USA) was used for all statistical analyses. Categorical variables were compared between groups by chi-square test and normally distributed continuous variables by Student's *t*-test. Verbal fluency task performance was compared between groups by the Mann-Whitney U-test. One-sample *t*-test was used to compare within-group task-related [oxy-Hb] vs. [oxy-Hb] = 0 (null hypothesis). Group differences in [oxy-Hb] were compared by covariance analysis with years of education as a covariate. The associations between [oxy-Hb] and PANSS scores were assessed by Spearman correlation tests. A *P* < 0.05 was considered statistically significant for all tests.

## Results

### Clinical Characters of Participants

Forty-seven patients with schizophrenia (SP group) and 29 age-, sex-, and body mass index-matched HCs (HC group) completed the study. The demographic and clinical characteristics of all participants are presented in Table 1. There were no significant group differences in basic demographic factors, while years of education was significantly lower in the SP group.

**Table 1:** Demographic and clinical characteristics of schizophrenia (SP) and healthy control (HC) groups.

	**SP (*n* = 47)**	**HC (*n* = 29)**	**t/*x*^**2**^**	***P***
Age	29.04 ± 7.32	29.2 ± 4.87	−0.085	0.933
Sex (male)	22	14	0.015	0.901
Years of education	11.98 ± 3.21	16.24 ± 1.70	−7.55	<0.001
Body Mass Index	23.81 ± 4.80	22.64 ± 2.99	1.313	0.194
Duration of illness, year	4.66 ± 2.61			
Age of onset, year	24.53 ± 6.73			
Time of admissions	3.09 ± 1.72			
PANSS				
Positive	13.72 ± 3.30			
Negative	12.55 ± 4.33			
General psychopathology	29.11 ± 5.00			
Chlorpromazine eq. dose (mg/day)	487.04 ± 186.59			
Mood stabilizers	21/47			
Benzodiazepines	18/47			

*Values expressed as mean ± standard deviation or number of participants*.

### VFT Performance

**Figure 3:**
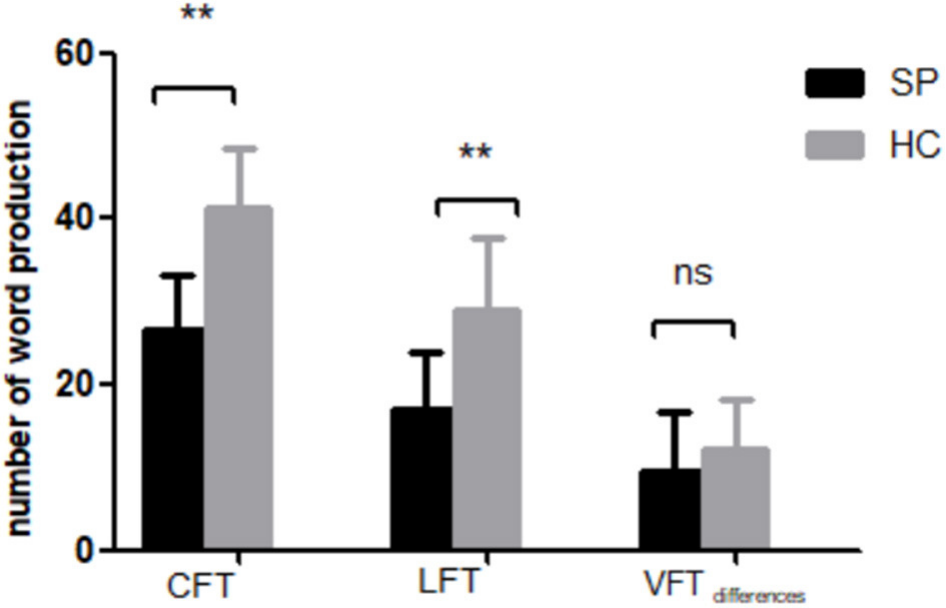
Performance of SP and HC groups on the category fluency task (CFT) and letter fluency task (LFT). VFT differences = n_CFT_-n_LFT_. **P < 0.001.

The SP group generated significantly fewer words than HCs during both the CFT [(26.62 ± 6.53) vs. (41.17 ± 7.42)] and LFT [(17.00 ± 6.84) vs. (28.93 ± 8.65)] (Figure 3). In contrast, the difference in task scores (CFT performance minus LFT performance) was similar in both groups [(9.62 ± 7.04) vs. (12.24 ± 5.97)]. Notably, CFT and LFT performance scores still differed between groups when education was included as a covariate (CFT: *F* = 35.42, *P* <0.001; LFT: *F* = 14.98, *P* <0.001; VFT performance differences: *F* = 3.12, *P* = 0.082). However, we found no significant associations between VFT task performances and [oxy-Hb] changes after FDR correction (FDR *P* <0.05).

### Cortical Activation Patters During VFTs

**Figure 4:**
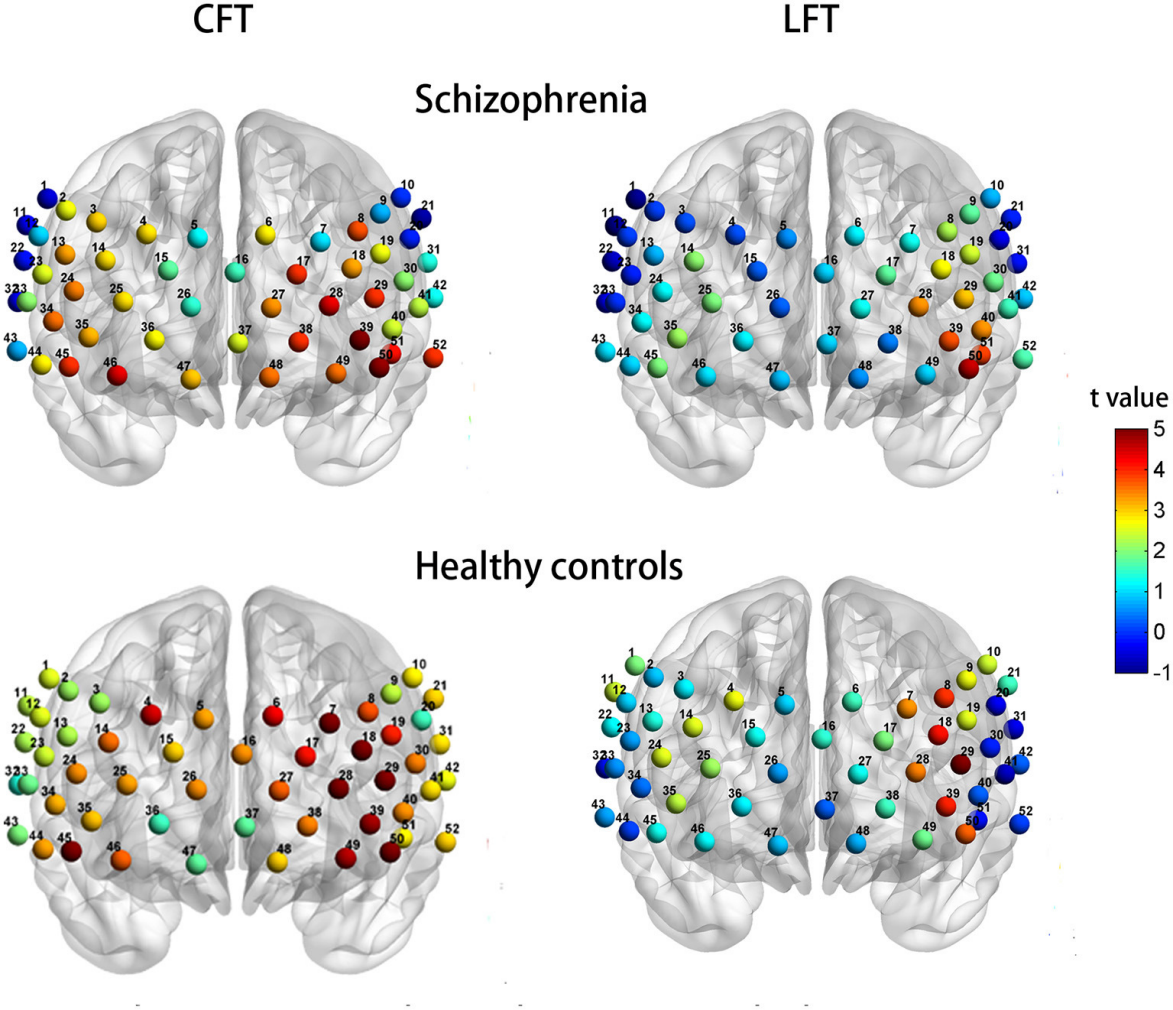
Patterns of cortical activation during the CFT **(left column)** and LFT **(right column)** for patients with schizophrenia **(top row)** and healthy controls **(bottom row)**.

The SP group exhibited significant activation of 33 channels (2–4, 6, 8, 13, 14, 17–19, 23–25, 27–29, 34–41, and 44–52; *t* = 2.379–5.496; FDR *P* <0.05; 15 channels over the left hemisphere and 17 channels over the right hemisphere, excluding medial channel 37) during the Chinese CFT. In contrast, only six channels showed significant activation during the LFT (28, 29, 39, 40, 50, and 51; *t* = 3.066–4.600; FDR *P* <0.05), all of which were over the left hemisphere (Figure 4, top row).

Healthy controls exhibited significant activation of 44 channels during the CFT (all except 2, 20, 32, 33, 36, 37, 43, and 47; *t* = 2.112–7.101; FDR *P* <0.05; 24 channels over the left hemisphere and 19 channels over the right hemisphere, ch16 excluded). Healthy controls also showed significant activation of seven channels during the LFT (7, 8, 18, 28, 29, 39, and 50; *t* = 3.408–5.972; FDR *P* <0.05), all of which were over the left hemisphere (Figure 4, bottom row).

### Group Differences in Activation Patterns

**Figure 5:**
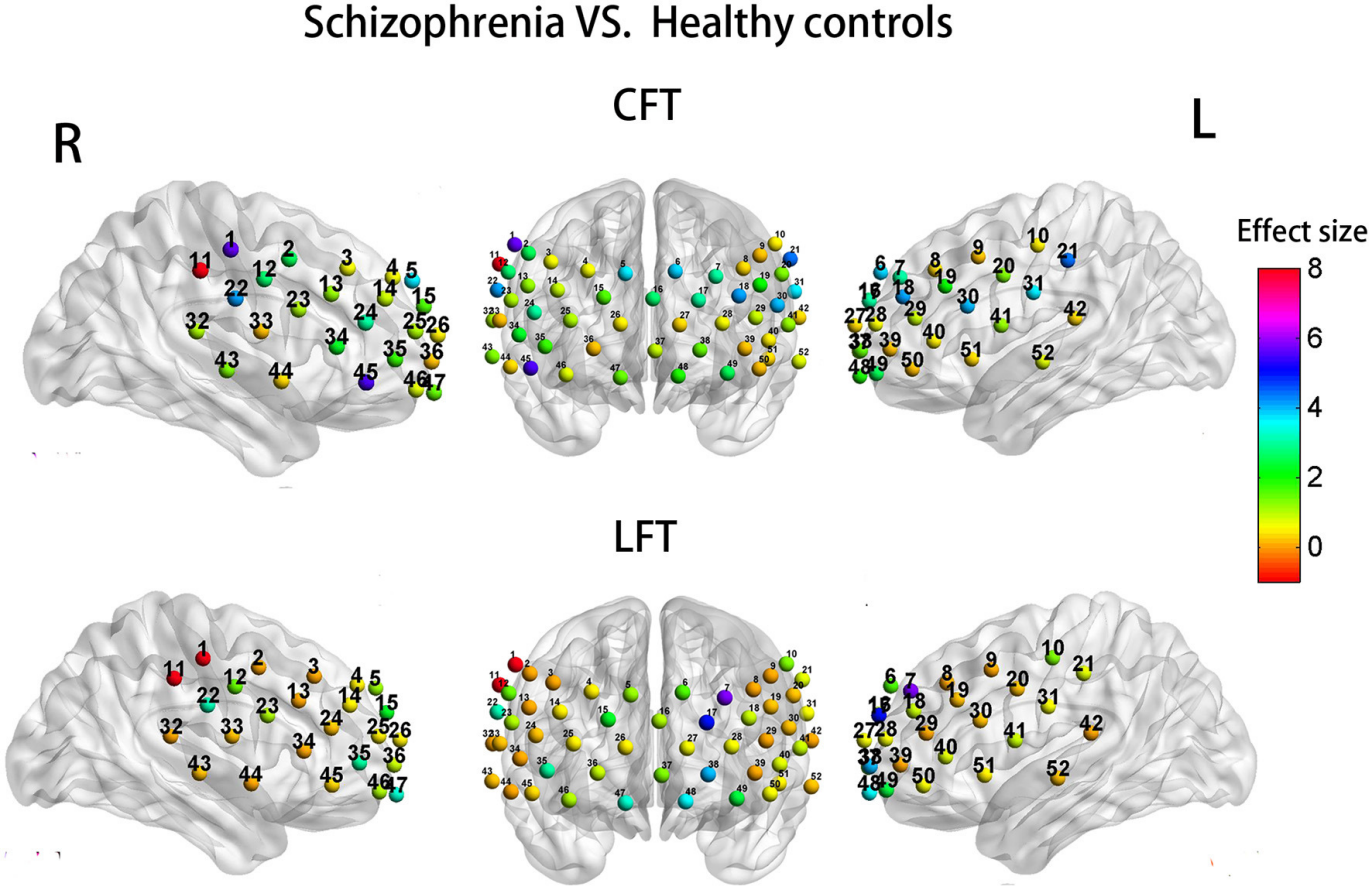
Group differences in task-related cortical activation during the CFT **(upper)** and LFT **(lower)**.

When years of education was included as a covariate, the SP group demonstrated significant hypoactivation relative to HCs at 7 channels during the CFT (1, 11, 18, 21, 22, 30, and 45; *F* = 4.047 −8.105; *P* = 0.006–0.048; Figure 5, upper row of images). However, there was no significant different with FDR set as 0.05. Similarly, during the LFT, when controlling for years of education, the SP group demonstrated significant hypoactivation relative to HCs at four channels (1, 7, 11, and 17) (*F* = 4.948–15.889; *P* = 0.000–0.030; Figure 5, lower row). After FDR correction, the significant different was only presented at channel 11 (FDR *P* <0.05).

### Correlational Analyses

Before the FDR correction, there were significantly positive correlations between activation strength and PANSS-Positive factor score during the CFT at channels 12 and 22 (over the right postcentral gyrus, PoCG; rho = 0.336 and 0.292, *P* <0.05). There were also significant positive correlations between activation strength during the CFT and PANSS-General psychopathology factor score at channel 23 (the right precentral gyrus, PreCG; rho = 0.413, *P* <0.01), channel 33 (right PoCG; rho = 0.373, *P* <0.01) as well as at channels 32 and 44 (the right superior temporal gyrus, STG; rho = 0.308 and 0.344, respectively, both *P* <0.05). However, these correlation coefficients were no longer significant after FDR correction (FDR *P* <0.05).

During the LFT, before the FDR correction, there were significantly positive correlations between activation strength and PANSS-Positive factor score at channel 1 (right PoCG) and channel 52 (left middle temporal gyrus, MTG) (rho = 0.327 and 0.340, respectively, *P* <0.05). In addition, there were significant negative correlations between activation strength and PANSS-Negative factor score at ten channels: channels 7, 18, and 24 (bilateral middle frontal gyrus, MFG), channels 29, 40, 41, 50, and 51 (all over the left inferior frontal gyrus, IFG), channel 11 (right supramarginal gyrus, SMG), and channel 31 (right PoCG) (rho = −0.293 to −0.364, all *P* <0.05). Again, however, these correlation coefficients were no longer significant after FDR correction (FDR *P* <0.05).

## Discussion

To our best knowledge, this is the first fNIRS study to compare cortical activation (as measured by [oxy-Hb]) between Chinese patients with schizophrenia and matched healthy controls during CFT and LFT performance. The main results can be summarized as follows: (1) the CFT induced wider cortical activation than the LFT in both groups, (2) patients showed reduced left hemisphere lateralization during the Chinese CFT, and (3) the CFT appears to be a more sensitive indicator of frontal-temporal dysfunction than the LFT in Chinese patients with schizophrenia.

### Patients With Schizophrenia Showed Similar Semantic and Phonemic Task Performance Deficits Compared to Healthy Controls

Patients with schizophrenia produced fewer words than controls during both the CFT and LFT, in accord with previous studies reporting pervasive language processing deficits in schizophrenia (34, 35). Moreover, both groups generated more words during the CFT than the LFT, indicating that the inherently superior semantic fluency observed in healthy individuals (36) was preserved in this patient cohort. Indeed, the difference in task score (CFT performance minus LFT performance) was not significantly different between the two groups, indicating that lower phonemic fluency or semantic fluency was not disproportionately greater in patients. This result is inconsistent with several studies reporting either a greater semantic deficit (11, 37–39) or phoneme deficit (9, 14) in patients with schizophrenia. However, most of these studies used English or Japanese VFTs, while our results are in line with a study of first-episode Chinese-speaking patients by Chou et al. (22). Thus, Chinese patients may show unique VFT performance deficits. A previous study (23) using fNIRS to examine cortical activation patterns during Chinese letter and semantic tasks also reported greater word production in healthy controls than patients with schizophrenia, but the authors did not compare performance between tasks, while other studies of Chinese schizophrenics used only one type of VFT (6, 12). Furthermore, years of education was not controlled in statistical analysis despite a potential impact on VFT performance. A study of Spanish patients with severe psychiatric disorders (40) found that CFT performance was less strongly influenced by years of education than LFT. Collectively, these results suggest that the CFT is superior to the LFT for evaluation of psychiatric disorders in Chinese speakers.

### Reduced Lateralization of Semantic Processing in Patients With Schizophrenia

In both groups, cortical activation ([oxy-Hb]) was higher and more extensive during the CFT than the LFT, in accordance with an fMRI study conducted in Chinese-speaking health controls (41). This may be explained by some level of phoneme analysis even when retrieving semantic information. Consistent with this notion, the aforementioned study (41) found activation of regions associated with phonemic fluency during the CFT but no significant activation of regions associated with semantic fluency during the LFT.

During the CFT, both patients with schizophrenia and controls exhibited significant activation of the bilateral frontal region and right superior temporal cortex, but left hemisphere activation was relatively more extensive and right frontotemporal activation more limited in healthy controls (i.e., controls showed greater left lateralization). In contrast, this group difference was not observed during the LFT, with both groups showing activation within overlapping regions of the left hemisphere, primarily in left ventrolateral prefrontal cortex. Similarly, a study of healthy individuals (42) also reported greater left hemispheric asymmetry of activation during a letter-cued task compared to a category-cued task. Thus, left lateralization was maintained in patients during the LFT, indicating that the LFT has less capacity to reveal cortical activation markers for schizophrenia.

These findings are in accord with a large number of neuroimaging studies reporting reduced left hemispheric laterality for language in schizophrenia patients (17, 43–46) compared to healthy populations (42, 47). However, Angrilli et al. (45) reported that reduced lateralization in Western patients with schizophrenia was specific for the phonological component of language. Therefore, lateralization of semantic and phonological processing between healthy controls and patients with schizophrenia may also differ between readers of Western and Eastern language script, possible due to the difference in semantic content.

### Differences in Regional Cortical Activation Patterns During VFTs Between Patients With Schizophrenia and Healthy Controls

Make mention of CFT, the schizophrenia patients exhibited no significant different reduced activation compared to controls after FDR correction, which was consistent with a previous fNIRS study (48) focusing on first episode schizophrenia and also used both VFTs as activation task but at odds with previous psychometric studies indicating that patients with schizophrenia have more severe semantic fluency deficits (7, 11, 39, 49–51). Similarly, with regard to LFT, the schizophrenia group showed significantly reduced activation in the right SMG (channel 11) compared to healthy controls. Chou et al. (48) suggested that LFT may be a more sensitive indicator of frontal dysfunction in schizophrenia than the CFT. However, when take the VFTs performance into consideration, the situation is vague. In accord with previous fNIRS studies (14, 15, 48, 50), we also found more word production during CFT than LFT in both groups. However, these studies indeed reported greater [oxy-Hb] activities during LFT than CFT. Remarkably, direct comparisons with previous neuroimaging studies of schizophrenia patients during VFTs may be misleading due to differences in task design, methodology, and study population. For instance, Quan et al. (12) found significantly lower [oxy-Hb] increases at 41 channels among patients during a Chinese-LFT compared to controls even after FDR correction. However, the study cohort was composed of chronic schizophrenia patients. In general, future studies lead to understanding the neural mechanisms of verbal fluency in Chinese schizophrenia patients were needed.

### Correlations Between [oxy-Hb] Changes and Clinical Symptom Severity

In the present study, we found no significant correlations between [oxy-Hb] changes and PANSS scores during either the CFT or LFT, possibly because the patients recruited were in a relatively stable disease stage. A previous fMRI study (17) reported that positive symptoms were associated with decreased left lateralization, suggesting that a relative increase in right hemispheric activity may predict psychosis. Similarly, an event-related potential study (45) and a previous NIRS study (52) both using phonological tasks found that decreased left lateralization was associated with positive symptoms. Moreover, a meta-analysis (53) concluded that reduced language lateralization could be a strong trait marker for auditory hallucinations (a positive symptom) among patients with schizophrenia. In contrast, Chou et al. (54) reported significant negative relationships between PANSS negative scores and activity in bilateral IFG and temporal regions during an LFT among patients with schizophrenia. They proposed that reduced gray matter volume in the frontal and temporal regions may contribute to the negative symptoms of schizophrenia. Consist with our results, Marumo et al. (15) also found no significant relationship between total PANSS score and [oxy-Hb] during either the CFT or LFT. However, the task design and statistical analysis methods differed from ours. Alternatively, Hori et al. (55) proposed that the association between functional laterality and clinical symptoms is strongly dependent on the activation task, so differences in task design may also contribute. Thus, potential correlations between [oxy-Hb] changes and clinical symptom severity are still uncertain. Larger scale studies recruiting participants at different clinical stages are needed to address this uncertainty.

### Limitations

This study has several limitations. First, the sample size was small, so other associations between cortical activity and schizophrenia symptoms may have been missed. Also, patients with schizophrenia show highly heterogeneous symptom expression, but the small sample precluded subgroup analysis. Second, all patients were currently taking antipsychotic drugs, which may have introduced further heterogeneity. However, we included only patients taking atypical antipsychotics based on evidence that these agents are more effective at maintaining cognitive function (56). In addition, a previous fNIRS study (57) reported that patients treated with atypical antipsychotics showed greater [oxy-Hb] during a VFT that patients treated with typical antipsychotics. Thus, the reduced cortical activation observed in schizophrenics may be unrelated to medication use. Moreover, we recruited schizophrenia patients at an early stage of the illness, and both task-dependent activation patterns and associations between these patterns and clinical parameters may differ from chronic patients (58). Last, all patients included in this study were relatively stable inpatients of a psychiatric hospital. Larger-scale studies including Chinese at different disease stages, under different treatment regimens, and from multiple centers are warranted to assess the general applicability of these fNIRS findings for patient evaluation.

## Data Availability Statement

The raw data supporting the conclusions of this article will be made available by the authors, without undue reservation.

## Ethics Statement

The studies involving human participants were reviewed and approved by the Ethics Committee of the Second Affiliated Hospital of Xinxiang Medical University. The patients/participants provided their written informed consent to participate in this study.

## Author Contributions

JL, XZ, and PL designed the study. JL and JM collected the data. JL, CS, and GY analyzed the experiment data. JL drafted the manuscript. JL, CS, KF, XZ, and PL approved the final version of the manuscript. All authors contributed to the article and approved the submitted version.

## Conflict of Interest

The authors declare that the research was conducted in the absence of any commercial or financial relationships that could be construed as a potential conflict of interest.

## Publisher's Note

All claims expressed in this article are solely those of the authors and do not necessarily represent those of their affiliated organizations, or those of the publisher, the editors and the reviewers. Any product that may be evaluated in this article, or claim that may be made by its manufacturer, is not guaranteed or endorsed by the publisher.
